# Novel unidirectional porous hydroxyapatite used as a bone substitute for open wedge high tibial osteotomy

**DOI:** 10.1007/s10856-014-5266-5

**Published:** 2014-07-05

**Authors:** Kenta Uemura, Akihiro Kanamori, Katsuya Aoto, Masashi Yamazaki, Masataka Sakane

**Affiliations:** 1 Department of Orthopaedic Surgery, Graduate School of Comprehensive Human Sciences, University of Tsukuba, 1-1-1 Tennodai Tsukuba, Ibaraki, 305-8575 Japan; 2 Department of Orthopaedic Surgery, Faculty of Medicine, University of Tsukuba, 1-1-1 Tennodai Tsukuba, Ibaraki, 305-8575 Japan

## Abstract

Purpose: The purpose of this study was to clinically and radiologically evaluate the availability, osteoconductivity, and resorption of a novel unidirectional porous hydroxyapatite (UDPHAp) used as an artificial substitute for open wedge high tibial osteotomy (OWHTO). Our hypothesis was that UDPHAp is a safe and useful bone substitute for OWHTO. Materials and methods: Seven patients (2 men and 5 women aged 34-72years) who underwent OWHTO and were followed up for more than 12months were retrospectively studied. After the osteotomy, the gap created was filled with UDPHAp(REGENOS® Kuraray Co.Ltd). Radiography and computed tomography(CT) were performed, and gap healing was assessed postoperatively. The Japanese Orthopaedic Association (JOA) knee score was determined pre- and post-operatively for clinical evaluation. Results: Neither gross displacement nor collapse of the UDPHAp block graft was observed within 12 months after surgery. Both radiographs and CT showed attenuation of lucency and increasing sclerosis over time. JOA score improved from 71.2 (65−80) to 95.8 (85−100). Conclusions: Short term results for OWHTO using UDPHAp was satisfactory. Clinical improvement of JOA scores were seen, besides osteogenesis was progressing in and around the artificial bone grafts.

## Introduction

High tibial osteotomy has become an effective surgical procedure for unicompartmental knee osteoarthritis or osteonecrosis of the knee [1, 2]. Open wedge high tibial osteotomy (OWHTO) is one option for the treatment of knee osteoarthritis and produces good clinical results [3, 4]. The gap left after the osteotomy is filled with an autologous, allogeneic, or artificial bone substitute or is left empty. Bioactive ceramics such as hydroxyapatite (HAp) and β-tricalcium phosphate (TCP) have been used as artificial bone substitutes for their high biocompatibility. HAp creates an apatite coat on its surface and fuses directly to natural cancellous bone. TCP naturally gets resorbed in vivo [5–8]. The purpose of this study was to clinically and radiologically evaluate the availability, osteoconductivity, and resorption of a novel unidirectional porous hydroxyapatite (UDPHAp; REGENOS^®^ Kuraray Co., Ltd., Okayama, Japan) used as an artificial substitute for OWHTO. Our hypothesis was that UDPHAp is a safe and useful bone substitute for OWHTO.

## Materials and Methods

**Fig. 1:**
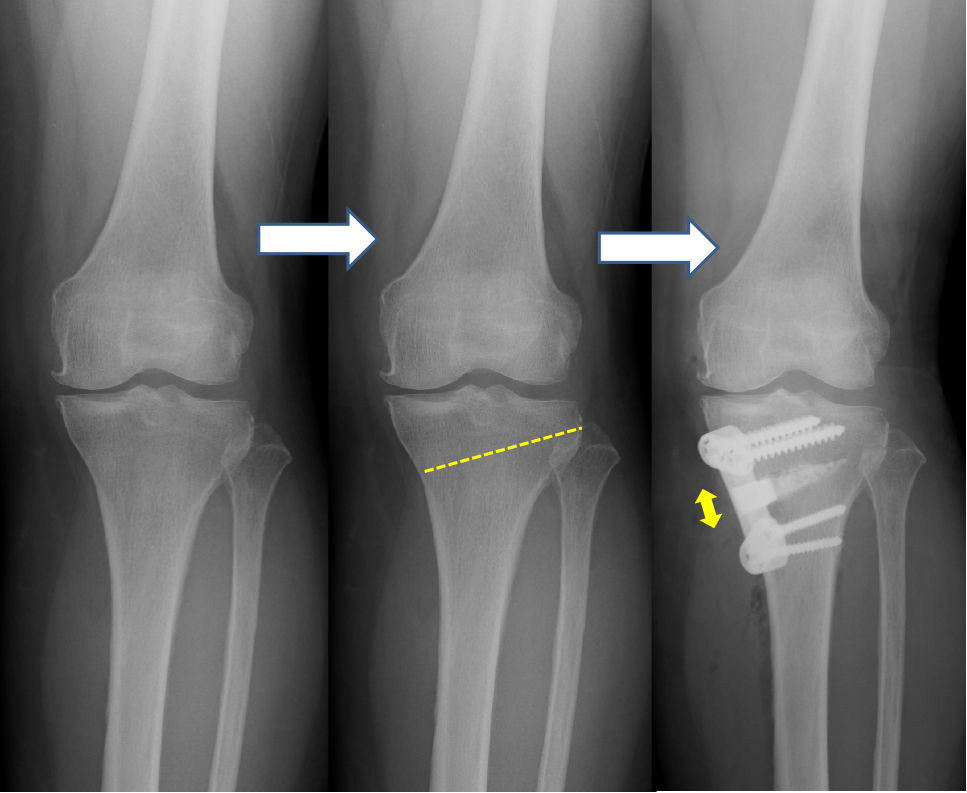
Osteotomy performed from the medial side of the tibia (*dashed line* osteotomy line). The osteotomy site was opened by the application of a valgus force that created a gap (⇔)

**Fig. 2:**
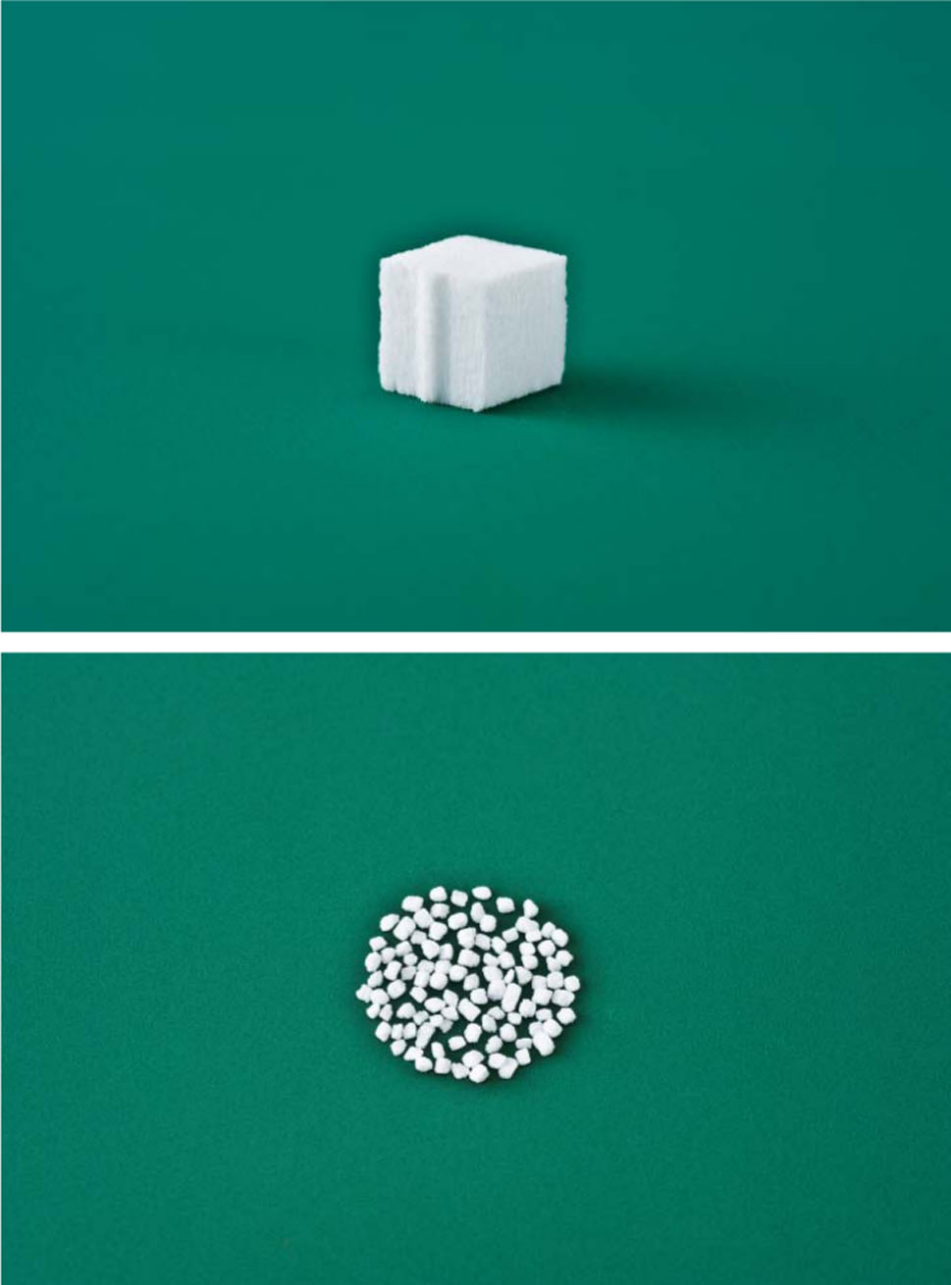
Pictures of the novel unidirectional porous hydroxyapatite (*above* block; *below* granules) provided by Kuraray Co., Ltd

**Fig. 3:**
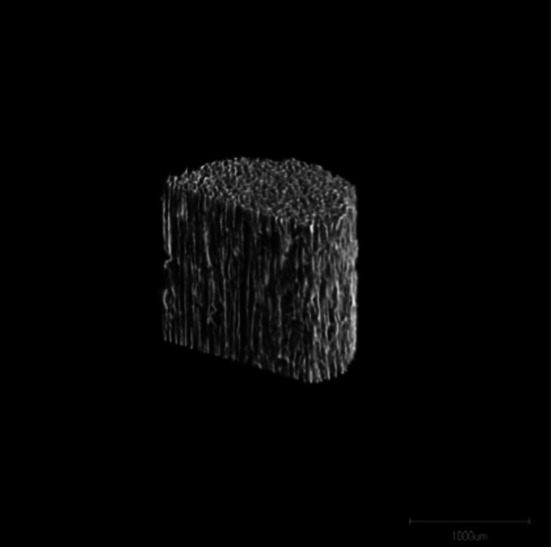
Three-dimensional micro-computed tomography image of the novel unidirectional porous hydroxyapatite (provided by Kuraray Co., Ltd.) showing unidirectional pores in the vertical direction and some interconnection toward the horizontal direction at the top

Seven patients (two men and five women aged 34–72 years) who underwent OWHTO and were followed up for 25 ± 7 (mean ± SD) months were retrospectively studied. Five patients had osteoarthritis of the medial compartment, one had spontaneous osteonecrosis of the medial femoral condyle, and one had an old lateral collateral ligament injury. As seen in Fig. 1, osteotomy was performed from the medial side of the proximal tibia laterally and proximally toward the head of the fibula. Biplanar osteotomy was used to increase rotational stability [9]. The osteotomy site was opened by the application of a valgus force to correct the varus deformity [10]. The correction angle was determined by the planning method according to Miniaci, and the opening height was calculated [11]. After osteotomy, the created gap was filled with UDPHAp (REGENOS^®^ Kuraray Co., Ltd.) and fixed with a Puddu plate (Arthrex Opening Wedge Plate System^®^; Arthrex Japan Co., Ltd., Tokyo, Japan). Two blocks of UDPHAp were cut to fit the gap and placed with the pores parallel to the tibial axis. UDPHAp granules (1 g total) were used to fill the tip of the gap (Fig. 2) and placed manually. This UDPHAp has a microstructure in which its cross-sectional oval pores (100–300 µm in diameter) penetrate through the material (Fig. 3).

Radiography and computed tomography (CT) were performed 1, 3, 6, and 12 months post-operatively to assess bone formation and resorption. On radiography, changes in the UDPHAp were followed in the anteroposterior (AP) view using the radiologic rating system reported by van Hemert WL [8]. Mechanical tibiofemoral angle (mTFA) was determined pre- and post-operatively using digital planning and standing full-limb AP radiographs. A negative value was given to knees in varus alignment [12]. In each CT series, we examined the same single slice in which the UDPHAp block was best visualized over time in both the sagittal and coronal planes. Using the method reported by Tanaka et al. [7], CT images parallel to the osteotomy plane were made and images at the centre were used to evaluate UDPHAp resorption and bone formation. The CT image data were divided into three areas (UDPHAp block, UDPHAp granules and hinged-cancellous bone) and CT attenuation values (in Hounsfield units: HU) of each area were analysed using the imaging software, Osirix (Pixmeo Inc., Geneva, Switzerland). The Japanese Orthopaedic Association (JOA) knee score was determined pre- and post-operatively for clinical evaluation. This score (on a scale of 0–100) consists of the following: pain on walking, 30; pain on ascending and descending the stairs, 25; range of motion, 35; joint swelling, 10.

Partial (1/3) weight-bearing was allowed 4–6 weeks postoperatively, while full weight-bearing was allowed 8–12 weeks post-operatively.

## Results

**Fig. 4:**
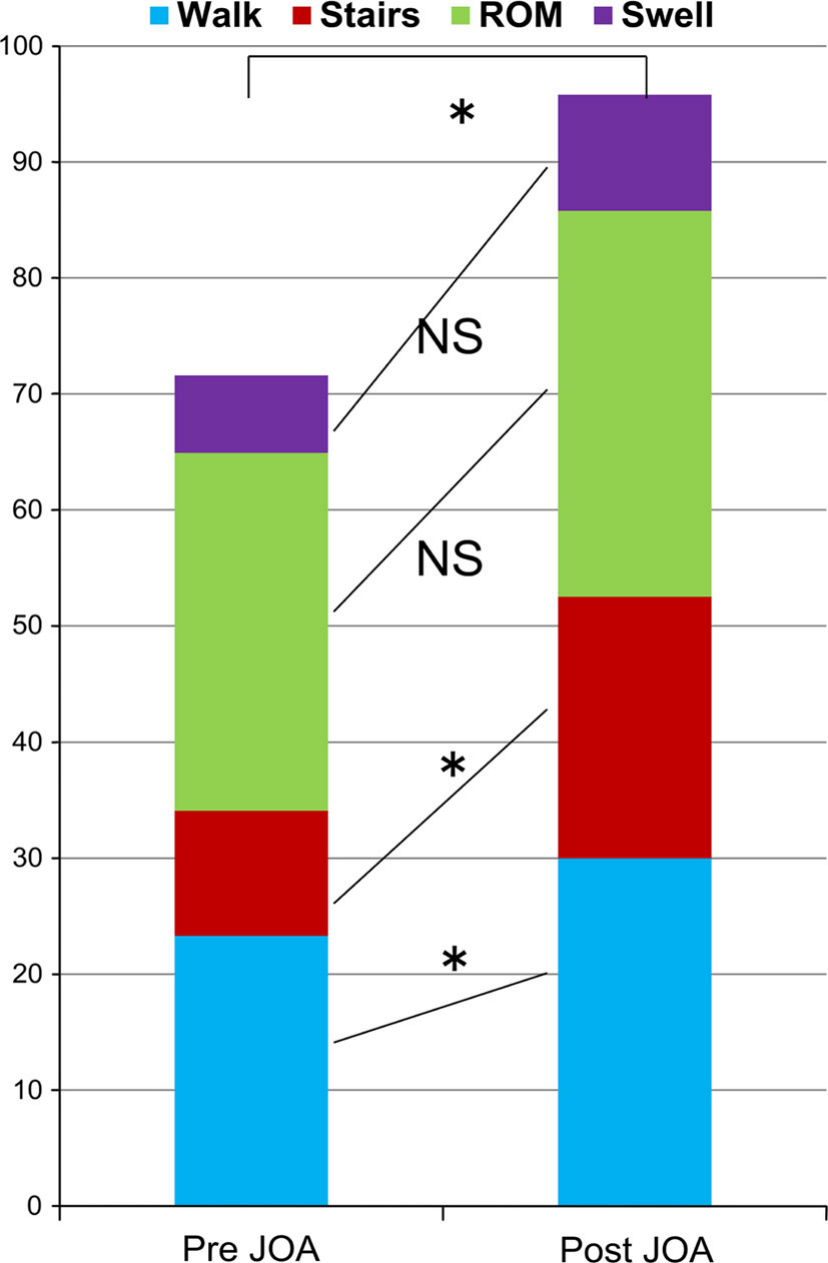
Change in Japanese Orthopaedic Association (JOA) scores. Wilcoxon signed-rank test, *P < 0.05 (n = 6)

We excluded one patient from the analysis who fell and broke his supracondylar femur. Activities of daily living improved in all patients, they complained of less pain in the knee, and no adverse effects of the bone graft were seen. The JOA score improved from 71.2 (range, 65–80) to 95.8 (85–100), while the mTFA improved from −8.5 (−6 to −14) to −0.3 (−3 to 3) degrees (Table 1; Fig. 4). There were no signs of infection or neuropathy.

**Table 1:** Pre- and post-operative evaluation data

	Pre-operation			12 m post-operation			
Case	ROM	JOA score	mTFA	ROM	JOA score	mTFA	Plate length(mm)
1.72 F OA	5–130	70 (25/10/25/10)	−14	3–140	90 (30/20/30/10)	−3	15.0
2.64 F OA	0–145	65 (25/5/30/5)	−6	0–145	85 (30/15/30/10)	−3	10.0
3.50 F OA	0–150	70 (25/10/30/5)	−8	0–150	100 (30/25/35/10)	−2	11.0
4.48 F OA	0–145	80 (25/15/35/5)	−6	0–145	100 (30/25/35/10)	−2	9.0
5.63 F SPONK	0–135	70 (25/10/30/5)	−9	0–150	100 (30/25/35/10)	−2	11.0
6.34 M LCL inj	0–140	75 (20/15/30/10)	−8	0–140	100 (30/25/35/10)	0	10.0

Japanese Orthopaedic Association (JOA) Knee Score (pain on walking, 30; pain on ascending and descending stairs, 25; range of motion, 35; joint swelling, 10)

ROM range of motion; mTFA mechanical femorotibial angle; OA Osteoarthritis

SPONK spontaneous osteonecrosis of the knee; LCL inj lateral collateral ligament injury

**Fig. 5:**
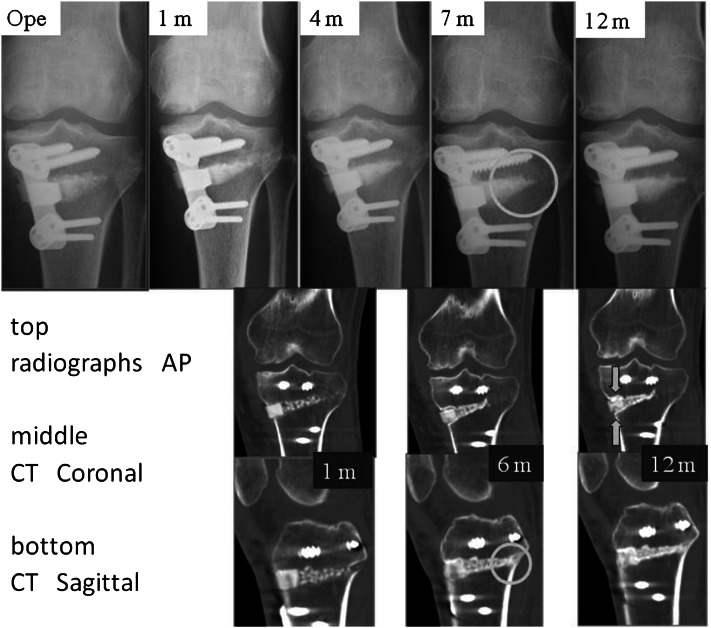
Case 5: 63-year old woman. Bone formation around the granules (*circle*); absorption of the novel unidirectional porous hydroxyapatite block (*arrows*)

**Fig. 6:**
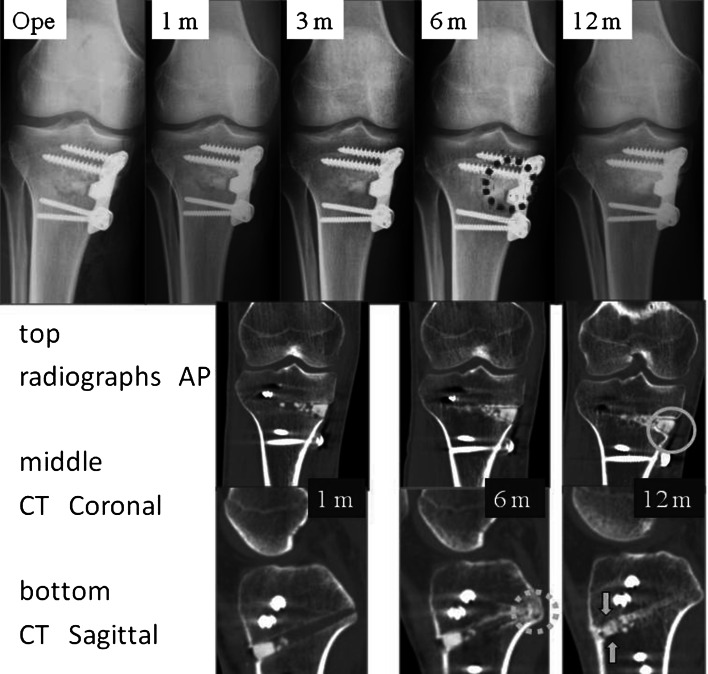
Case 6: 34- year old man. Bone formation around the granules (*dashed circle*); absorption of the novel unidirectional porous hydroxyapatite block (*arrows*); lucent lines between the graft and adjacent bone (*circle*)

Neither gross displacement nor collapse of the UDPHAp block graft was observed within 12 months after surgery. Both radiographs and CT images showed attenuated lucency and increasing sclerosis over time. Bone formation was seen chronologically in all cases, especially in the posteromedial section where the granules were placed (Fig. 5, 6). We slightly modified the grading of van Hemert; as shown in Table 2, the bone union rate progressed gradually, and no lucent line was visible on the radiographs after 12 months.

**Table 2:** Progress in radiological bone healing at 1, 3, 6, and 12 months after surgery

Grade	1 month	3 months	6 months	12 months
1	100 % (n = 6)			
2		50 % (n = 3)		
3		50 % (n = 3)	67 % (n = 4)	
4			33 % (n = 2)	100 % (n = 6)
5				

Grade 1 is a vascular phase with clear distinction between the hydroxyapatite and bone. Grade 2 is a calcification phase with blurred distinction. Grade 3 is an osteoblastic phase with slightly visible distinction. Grade 4 is a consolidation phase with no lucent signs despite recognizable osteotomy. Grade 5 is full reformation with no sign of osteotomy

**Fig. 7:**
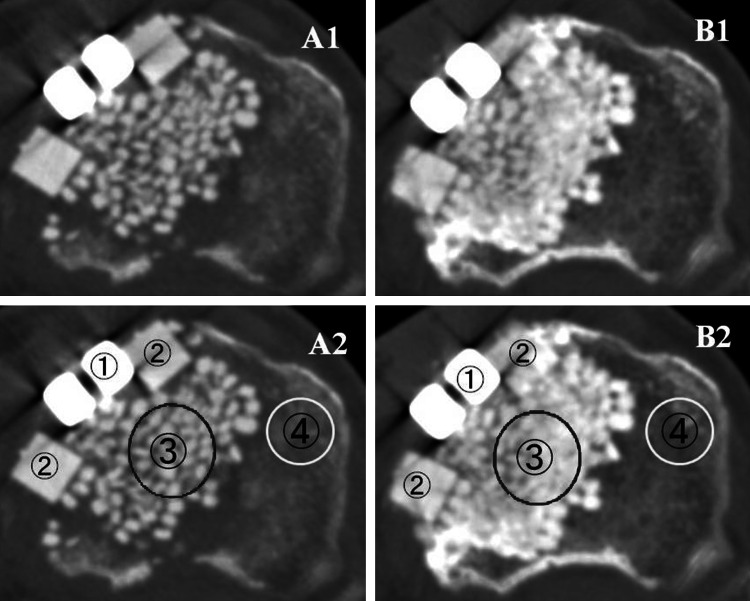
Case 5: 63-year old woman. Computed tomography (CT) images showing the centre of the osteotomy plane at 1 month (A1, A2) and 12 months (B1, B2). The mean CT values (HU) of the area implanted with the novel unidirectional porous hydroxyapatite block (➁), the area implanted with granules (➂), hinged-cancellous bone (➃) changed from 950, 662, and 172, to 798, 951, and 186, respectively. ➀ indicates the area of the metal plate

Resorption of the UDPHAp block was studied using CT (Fig. 7). The mean CT values (HU) of the area implanted with UDPHAp blocks at 1 and 12 months were 936 ± 31 and 838 ± 50, respectively.

The mean CT values of the area implanted with UDPHAp granules at 1 and 12 months were 629 ± 37 and 856 ± 159, respectively. The mean CT values of cancellous bone at 1 and 12 months were 128 ± 57 and 156 ± 27, respectively.

## Discussion

The hypothesis that UDPHAp is a safe and useful bone substitute for OWHTO was proven in this 1 year post-operative study.

It was predicted that large stress will be applied on the plate and around the osteotomy site when weight bearing is started after OWHTO [13]. Rigid locking plates that are much more stable than the previously used implants have recently been used. The two most commonly used locking plates these days are the Tomofix plate (Synthes K.K., Tokyo, Japan) and the Puddu plate (Arthrex Japan Co., Ltd.). In a biomechanical study, a long locking plate had a greater effect on primary stability than a short spacer plate [14]. The Tomofix is a long locking plate, but it has a bulky design and can cause patient discomfort when placed on the front side of the tibia near the skin [9]. For this reason, plate removal is necessary in almost every case. Since we were treating relatively small Japanese patients (body size compared with German) in this study, we chose the Puddu plate.

Many research teams have suggested that the use of supporting material could protect against collapse of the osteotomy opening and accelerate bony healing without causing any disadvantages for patients and that it was a better option than leaving the gap open [8–10, 13, 15, 16]. The most suitable method for filling the opened gap in OWHTO has yet to be identified. Autologous bone grafting has procurement morbidities. The use of allografts is not allowed in Japan due to disease transmission problems. [7] One study, in which the gap was left empty, reported complications such as implant breakage [17]. Artificial bone materials have recently been used as alternatives to autografts and allografts.

Artificial bone substitutes are needed that feature the following: sufficient strength as a supporting structure; the availability of intra-operative shape adjustments; high affinity for bone tissue; ability to promote bone formation; and ability to decompose and be resorbed in vivo [18].

Porous HAp, which lacks interconnecting pores and has a porosity of approximately 40 %, has been said to be suitable for use as a bone-graft substitute in medial OWHTO. On autopsy specimens after OWHTO with the use of a porous HAp wedge, >70 % of the pores were filled with newly generated bone [15, 19]. TCP has been reported to be suitable as a bone substitute because of its favourable biocompatibility, osteoconduction, and resorption properties [7, 8, 13, 16]. For initial compression strength, a TCP block with 75 % porosity is inadequate for weight bearing until bone incorporation occurs [7]. In a 6-year study, TCP with 75 % porosity was completely resorbed, although 1/3 of TCP with 60 % porosity remained [5]. The present novel UDPHAp has a porosity of 75 % and an initial compression strength parallel to the unidirectional pores of approximately 14 MPa, making it sufficiently strong to bear the load applied to the tibia. UDPHAp can easily be cut by a saw to fit the gap. As would be predicted from the unidirectional pores, cellular and blood migration and invasion occur rapidly. All margins of the block and granules became unclear, suggesting surrounding osteogenesis. On CT, UDPHAp blocks showed a gradual decrease in HU score, suggesting affinity with the bone and decomposition in vivo. Osteogenesis was seen around the granules, and after its sclerotic change, its HU score increased.

Watanabe et al. [20] performed a tibial wedge osteotomy in canines and implanted UDPHAp within the bone defect. They made a wedge-shaped bone defect and implanted a press-fittable UDPHAp with no gap. On radiological and histological examination at 12 weeks, UDPHAp showed bony consolidation and direct bonding with the osteotomy site. We performed osteotomy and opened it with a valgus force. Instead of a press-fittable wedge, we used blocks and granules. The unidirectional pores were positioned parallel to the loading direction. Lucent lines were visible in some cases between the graft and adjacent bone, possibly because fibrous tissues had entered the gap. Clinical assessment focused on bony healing, and better outcomes were expected if the blocks were cut carefully enough to fill the gap.

UDPHAp has a high compression strength parallel to the unidirectional pores of 14 MPa, whereas other HAp products have 2–10 MPa [18]. As good clinical results have been reported with this initial strength, we predict that post-operative weight bearing can be allowed earlier using UDPHAp.

## Conclusions

The short-term results of OWHTO using UDPHAp were satisfactory with no harmful effects and the correction angle was maintained. JOA scores improved, and the imaging results indicated that osteogenesis was progressing in and around the artificial bone grafts.

## References

[1] Coventry MB (1993). Proximal tibial osteotomy: a critical long-term study of eighty-seven cases. J Bone Joint Surg Am.

[2] Marti CB (2000). Spontaneous osteonecrosis of the medial compartment of the knee: a MRI follow-up after conservative and operative treatment, preliminary results. Knee Surg Sports Traumatol Arthrosc.

[3] Hernigou P (1987). Proximal tibial osteotomy for osteoarthritis with varus deformity. J Bone Joint Surg Am.

[4] Spahn G (2002). Primary stability of various implants in tibial opening wedge osteotomy: a biomechanical study. J Orthop Sci..

[5] Tanaka T, et al. A novel evaluation system to monitor bone formation and β-tricalcium phosphate resorption in opening wedge high tibial osteotomy. Knee Surg Sports Traumatol Arthrosc. 2014. doi:10.1007/s00167-014-2870-3. 10.1007/s00167-014-2870-3 PMC4471387 24497055

[6] Murakoshi T (2012). Characterization of mechanical properties and bioactivity of hydroxyapatite/β-tricalcium phosphate composites. J Jpn Soc Compos Mater..

[7] Tanaka T (2008). Bone formation and resorption in patients after implantation of β tricalcium phosphate blocks with 60% and 75% porosity in opening-wedge high tibial osteotomy. J Biomed Mater Res B.

[8] Van Hemert WL (2004). Tricalcium phosphate granules or rigid wedge preforms in open wedge high tibial osteotomy: a radiological study with a new evaluation system. Knee..

[9] Aryee S (2008). Do we need synthetic osteotomy augmentation materials for opening-wedge high tibial osteotomy. Biomaterials.

[10] Hernigou P (2001). Opening wedge tibial osteotomy with acrylic bone cement as bone substitute. Knee..

[11] Pape D (2007). Preoperative planning for high tibial osteotomies. Oper Tech Orthop..

[12] Chang CB (2010). What should be considered in using standard knee radiographs to estimate mechanical alignment of the knee?. Osteoarthr cartil.

[13] Takeuchi R (2010). In vitro stability of open wedge high tibial osteotomy with synthetic bone graft. Knee..

[14] Agneskirchner JD (2006). Primary stability of four different implants for opening wedge high tibial osteotomy. Knee Surg Sports Traumatol Arthrosc.

[15] Koshino T (2003). Medial opening-wedge high tibial osteotomy with use of porous hydroxyapatite to treat medial compartment osteoarthritis of the knee. J Bone Joint Surg Am.

[16] Takeuchi R (2009). Medial opening wedge high tibial osteotomy with early full weight bearing. Arthroscopy.

[17] Miller BS (2009). Complications after medial opening wedge high tibial osteotomy. Arthroscopy.

[18] Myoui A (2011). The history of artificial bones and its latest design and concept (in Japanese) Jpn. J Artif Organs..

[19] Koshino T (2001). New bone formation around porous hydroxyapatite wedge implanted in opening wedge high tibial osteotomy in patients with osteoarthritis. Biomaterials.

[20] Watanabe A (2010). Novel unidirectional porous hydroxyapatite used as a bone substitute for tibial wedge osteotomy in canines. Biomater Res..

